# A fast method for calculating reliable event supports in tree reconciliations via Pareto optimality

**DOI:** 10.1186/s12859-015-0803-x

**Published:** 2015-11-14

**Authors:** Thu-Hien To, Edwin Jacox, Vincent Ranwez, Celine Scornavacca

**Affiliations:** 1 ISEM - Université de Montpellier, CNRS, IRD, EPHE, Place Eugène Bataillon, Montpellier, 34392 France; 2 Montpellier SupAgro, UMR AGAP, 2 Place P. Viala, Montpellier, 34060 France; 3 Institut de Biologie Computationnelle (IBC), Montpellier, 34095 France

**Keywords:** Tree reconciliation, Gene evolution, Phylogenetics, Parsimony, Supports

## Abstract

**Background:**

Given a gene and a species tree, reconciliation methods attempt to retrieve the macro-evolutionary events that best explain the discrepancies between the two tree topologies. The DTL parsimonious approach searches for a most parsimonious reconciliation between a gene tree and a (dated) species tree, considering four possible macro-evolutionary events (speciation, duplication, transfer, and loss) with specific costs. Unfortunately, many events are erroneously predicted due to errors in the input trees, inappropriate input cost values or because of the existence of several equally parsimonious scenarios. It is thus crucial to provide a measure of the reliability for predicted events. It has been recently proposed that the reliability of an event can be estimated via its frequency in the set of most parsimonious reconciliations obtained using a variety of reasonable input cost vectors. To compute such a support, a straightforward but time-consuming approach is to generate the costs slightly departing from the original ones, independently compute the set of all most parsimonious reconciliations for each vector, and combine these sets a posteriori. Another proposed approach uses Pareto-optimality to partition cost values into regions which induce reconciliations with the same number of DTL events. The support of an event is then defined as its frequency in the set of regions. However, often, the number of regions is not large enough to provide reliable supports.

**Results:**

We present here a method to compute efficiently event supports via a polynomial-sized graph, which can represent all reconciliations for several different costs. Moreover, two methods are proposed to take into account alternative input costs: either explicitly providing an input cost range or allowing a tolerance for the over cost of a reconciliation. Our methods are faster than the region based method, substantially faster than the sampling-costs approach, and have a higher event-prediction accuracy on simulated data.

**Conclusions:**

We propose a new approach to improve the accuracy of event supports for parsimonious reconciliation methods to account for uncertainty in the input costs. Furthermore, because of their speed, our methods can be used on large gene families. Our algorithms are implemented in the ecceTERA program, freely available from http://mbb.univ-montp2.fr/MBB/.

**Electronic supplementary material:**

The online version of this article (doi:10.1186/s12859-015-0803-x) contains supplementary material, which is available to authorized users.

## Background

The evolutionary history of a gene family often differs from the history of the species containing those genes due to macro-evolutionary events other than speciation. Reconciliation methods compare gene trees with a species tree in order to recover these events. The DTL reconciliation model [1–4] accounts for three types of events: gene duplications ($\mathbb {D}$), losses ($\mathbb {L}$), and transfers ($\mathbb {T}$). This model is typically used in a parsimony framework, which searches for the reconciliations that minimize the overall cost, given a cost vector specifying the costs for $\mathbb {D}, \mathbb {T}$ and $\mathbb {L}$ events. Unfortunately, ensuring the time-consistency of gene transfers, i.e., satisfying the chronological constraints among nodes of the species tree that are involved in transfer events, is NP-hard [4, 5]. However, if the internal nodes of the species tree are ordered, i.e. using a dated species tree, the problem can be efficiently solved [1, 6]. Although there can be an exponential number of optimal reconciliations for a given cost vector, the *DTL-graph* can be used to represent them compactly [7].

Some of the events predicted by reconciliations methods may not be reliable due to potential inaccuracies in the input trees and the inherent imprecision of the input costs. Thus, it is necessary to estimate confidence values, or *supports*, for each predicted event. Supports can be defined, for example, as the frequency of an event over a space of alternative solutions.

In [7–9], the support of an event is defined as the frequency of the reconciliations containing it over all reconciliations in the solution space. In [7, 8], the DTL-graph was used to compute event supports in this sense over the set of all parsimonious solutions (for one cost vector in [7] and for several ones in [8]). Moreover, in [8], the authors used these event supports to identify the *median reconciliation*, that is the reconciliation that minimizes the sum of event-based distances between itself and all other parsimonious alternatives (here, the event-based distance between two reconciliations is defined as the number of events contained in one but not in the other). Bansal et al. [9] presented another way to address the problem, suitable for non-dated species trees as well as dated ones: they designed a method which samples the space of parsimonious reconciliations uniformly at random and then counts the frequency of each event within all sampled solutions.

However, the approaches presented in [7, 9] only focus on one cost vector; though, it is often difficult to know the appropriate cost vector, and the solutions can be very sensitive to the input costs. In [8], the authors used a time-consuming approach that samples the space of cost vectors around the input one and then apply the algorithm presented in [7] to compute a DTL-graph for each sample. Libeskind-Hadas et al. [10] presented a method to estimate the sensibility of a parsimonious reconciliation with respect to the input costs. They use *Pareto-optimal* event count vectors to partition the cost space into regions such that the costs in the same region induce reconciliations with the same number of DTL events. These regions are then used to compute events supports, defined as the fraction of regions having an event in all (or one of, depending on the option used) their reconciliations. However, for the support measure to be reliable, the events should be recovered using biologically realistic costs, which can vary with respect to the phylum we are interested in (e.g., in Mammalian, duplications are much more frequent than transfers, whilst transfers are predominant in the evolution of Bacteria).

But, when analyzing a realistic cost space with the method in [10], we observe that, often, a small number of regions, e.g. in many cases only one, is returned; thus, the corresponding supports can only be either 0 or 1 and are almost useless to filter out events. On the other hand, a larger cost space can account for more regions, but the event supports may be unreliable since they consider events retrieved using unrealistic costs (we will see in the Results section that this is indeed the case). Moreover, the method in [10] does not generate median reconciliations. It was shown in [8] that the median reconciliation has better event predictions than random ones.

Here, we use the definition of an event support given in [7, 8] and extend the work in [7] by taking into account several cost vectors. Rather than using the time-consuming sampling method in [8], we construct the space of all solutions corresponding to the input cost range and then compute the event supports over this space. As we shall see, our algorithm also makes use of Pareto-optimal reconciliations, which are efficiently computed via an extended version of the DTL-graph. This has the advantage of efficiently taking into account all relevant parsimonious reconciliations – even those that are parsimonious for very few event cost combinations and that are thus unlikely to be discovered by an ad hoc choice or a random sampling of event costs [10].

The approaches presented in [7–9] only consider optimal parsimonious reconciliations, even though the real gene evolution might not be optimal. In this paper, we also present a method for computing near optimal reconciliations that allows a tolerance *ε* for the overall reconciliation cost. Our methods are faster than the one presented in [10], substantially faster than the sampling-costs approach presented in [8], and have a higher event prediction accuracy on simulated data than both methods.

### Basic notations

Let *T* be a binary rooted tree where only leaf nodes are labeled. We denote by *V*(*T*),*E*(*T*), *r*(*T*),*L*(*T*), and $\mathcal {L}(T)$ respectively the sets of nodes and edges, the root node, the set of leaf nodes, and the set of species labeling the leaves of *T*. If *u* is a leaf node, we denote by *s*(*u*) the species that labels *u*, and if *u* is not a root we denote by *u*_*p*_ its parent and by (*u*_*p*_,*u*) the edge connecting *u*_*p*_ and *u*. Note that, in a rooted tree, all edges are directed away from the root. For any node *u* of *T*, *T*_*u*_ is the subtree of *T* rooted at *u*.

**Fig. 1:**
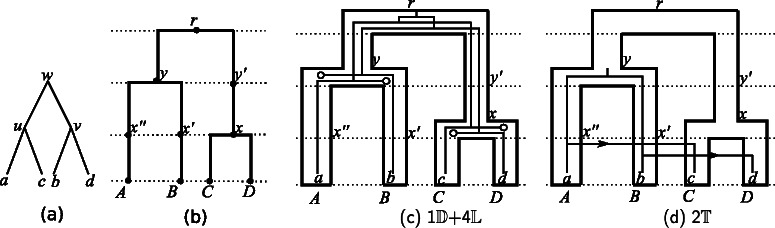
A gene tree (**a**) and the subdivision of a dated species tree (**b**). Two reconciliations between the trees (**a**) and (**b**) are shown in (**c**, **d**): each ∘ corresponds to a loss, each arrow indicates a transfer event from the donor to the receiver species

A tree *T* is *dated* if it is associated with a *time function* $\theta :V(T) \to \mathbb {R}^{+}$ such that *θ*(*u*)=0 for every leaf *u* and *θ*(*u*)<*θ*(*v*) if *u* is a strict descendant of *v*. Given a dated tree *T* and a positive time *t*, we denote as *V*_*t*_(*T*) the set of nodes of *T* at time *t*. In this context, we are only interested in the order of internal nodes. Hence the time function consists of integer values: 0 (for the leaves), 1, 2, … We can derive the *dated subdivision tree* *T* ^′^ from a dated tree *T* by adding, on each edge (*u*_*p*_,*u*)∈*E*(*T*) such that there exists *z*∈*V*(*T*) with *θ*(*u*)<*θ*(*z*)<*θ*(*u*_*p*_), a new node *y* with *θ*(*y*)=*θ*(*z*). An internal node *u* is said to be *artificial* if it has only one child, denoted by *u*_1_; otherwise we call it a *speciation* node and we denote its two children by *u*_1_ and *u*_2_. The time interval between a node of *T* ^′^ and its parent is called a time slice. For example, Fig. 1 b represents the subdivision of the tree ((*A,B*),(*C,D*)) in which *θ*(*x*)=1,*θ*(*y*)=2 and *θ*(*r*)=3. Artificial nodes are used to ensure the time-consistency of gene transfers efficiently (more on this below).

A *species tree* *S* is a rooted binary tree such that each leaf represents an extant species and there is a bijection between *L*(*S*) and $\mathcal {L}(S)$. A *gene tree* *G* is a rooted binary tree such that each leaf corresponds to a contemporary gene and is labeled by the species that contains it.

In this paper, we will use *G* to denote a gene tree, and *S* ^′^ to denote the subdivision of a dated species tree *S* such that $\mathcal {L}(G)\subseteq \mathcal {L}(S)$.

Finally, given an order list *L*, we denote by *L*_*i*_ the *i* ^*th*^ element of *L*.

### Reconciliation

**Fig. 2:**
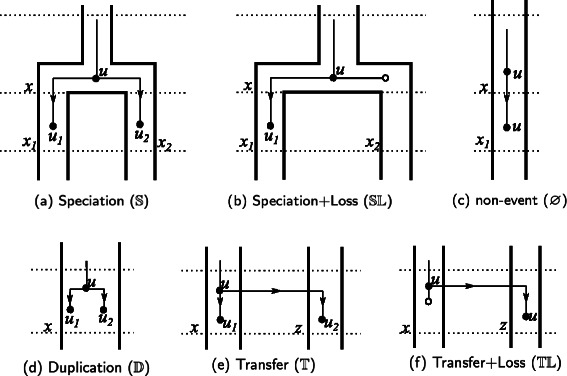
An illustration of the basic events described in Definition 1

In this paper, we build on the reconciliation model accounting for duplication, loss and transfer events introduced in [1]. In this model, the time-consistency of gene transfers is ensured by constraining each transfer event to happen between a donor and a receiver species in the same set *V*_*t*_(*T*) and hence in the same time slice. The seven basic events considered by this model are depicted in Fig. 2: speciation ($\mathbb {S}$), duplication ($\mathbb {D}$), transfer ($\mathbb {T}$), contemporary ($\mathbb {C}$), transfer+loss $(\mathbb {TL}$) and speciation+loss $(\mathbb {SL}$) events, plus the additional no event ($\varnothing $). $\mathbb {S}, \mathbb {D}$ and $\mathbb {T}$ events are self-explanatory. A $\varnothing $ event denotes that a gene crosses a time boundary, with no other event happening. A contemporary event $\mathbb {C}$ associates a leaf *u* of *G* with a leaf *x* of *S* ^′^ such that *s*(*u*)=*s*(*x*). A duplication followed immediately by a loss, i.e. a $\mathbb {DL}$ event, can occur an arbitrary number of times in a reconciliation, making the solution space infinite. To prevent this, loss events are never considered alone but are always coupled with either a speciation or a transfer event. Thus, an $\mathbb {SL}$ event is a speciation where the gene is lost in one of the two derived species, while a $\mathbb {TL}$ event is a $\mathbb {T}$ event where the transferred gene is not kept in the descendants of the donor species. Note that the models of [9–11] only consider $\mathbb {SL}$ events and not $\mathbb {TL}$ events. Consecutive $\mathbb {TL}$ events are not allowed in order to prevent the solution space from being infinite. Note that $\mathbb {D}\mathbb {L}$ events and consecutive $\mathbb {TL}$ cannot happen in parsimonious reconciliations. $\mathbb {S}, \mathbb {D}, \mathbb {T}$ are called *birth-events*, since they produce new lineages.

A reconciliation *α* is defined as a function that maps each vertex *u* of *G* onto an order sequence of nodes (*x*_1_,⋯,*x*_*k*_) of *S* ^′^ and such that the mappings of *u* and its children satisfy some *biologically-dictated* constraints [1]. In Definition 2, we express these constraints via the function *postlist* defined below, which, for every *u*∈*V*(*G*), *x*∈*V*(*S* ^′^) and $e \in \{\mathbb {S},\mathbb {D},\mathbb {T},\mathbb {SL},\mathbb {TL},\mathbb {C},\varnothing \}$, defines all possible mappings of the children of *u*, denoted *u*_1_,*u*_2_, if *e* is a birth-event, or all possible next mappings of *u* otherwise (see Fig. 2). Hence, *p* *o* *s* *t* *l* *i* *s* *t*_*e*_(*u,x*) is a set of sets of pairs if $e\in \{\mathbb {S},\mathbb {D},\mathbb {T}\}$, and a set of pairs if $e\in \{\mathbb {SL}, \mathbb {TL},\varnothing \}$.

#### **Definition1**.

For every *u*∈*V*(*G*)∖*L*(*G*), *x*∈*V*(*S* ^′^):

1. $postlist_{\mathbb {S}}(u,x)= \{\{(u_{1},x_{1}),(u_{2},x_{2})\}$, {(*u*_1_,*x*_2_),(*u*_2_,*x*_1_)}} if *x* is a speciation node and *u* is not a leaf, otherwise *∅*.
2. $postlist_{\mathbb {D}}(u,x)= \{\{(u_{1},x),(u_{2},x)\}\}$ if *u* is not a leaf, otherwise *∅*.
3. $postlist_{\mathbb {T}}(u,x)=\bigcup \limits _{z\neq x, \theta (z)=\theta (x)} \{\{(u_{1},x), (u_{2},z)\}, \{(u_{2},x), (u_{1},z)\}\}$, if *u* is not a leaf, otherwise *∅*.
4. $postlist_{\varnothing }(u,x)=\{{(u,x_{1})\} }$ if *x* is an artificial node, otherwise *∅*.
5. $postlist_{\mathbb {SL}}(u,x)=\{(u,x_{1}),(u,x_{2})\} $ if *x* is a speciation node, otherwise *∅*.
6. $postlist_{\mathbb {TL}}(u,x)={\bigcup \limits _{z\neq x, \theta (z)=\theta (x)}\{(u,z)\}}$.

The definition of a reconciliation can be adapted from [1] and [7] by using *postlist* as follows.

#### **Definition2** (Reconciliation).

Let *α* be a function mapping each node *u* of *G* onto an ordered sequence of nodes of *S* ^′^. We denote by *α*_*ℓ*_(*u*) the last element of the sequence *α*(*u*). For all *u*∉*L*(*G*), let *p* *o* *s* *t*_*α*_(*u*,*α*_*i*_(*u*)) be (*u*,*α*_*i*+1_(*u*)) if *i*≠*ℓ*, and {(*u*_1_,*α*_1_(*u*_1_)),(*u*_2_,*α*_1_(*u*_2_))} otherwise. Then, *α* is said to be a *reconciliation* between *G* and *S* ^′^ if and only if, for each pair of nodes *u* of *G* and *x*=*α*_*i*_(*u*) of *S* ^′^, one of these conditions holds:

1. *i*=*ℓ*, *u*∈*L*(*G*), and *x*∈*L*(*S* ^′^), *s*(*x*)=*s*(*u*); $(\mathbb {C}$ event);
2. *i*=*ℓ*, *u*∉*L*(*G*), and there exists exactly one event type $e\in \{\mathbb {S},\mathbb {D},\mathbb {T}\}$ such that *p* *o* *s* *t*_*α*_(*u,x*)∈*p* *o* *s* *t* *l* *i* *s* *t*_*e*_(*u,x*);
3. *i*≠*ℓ*, and there exists exactly one event type $e\in \{\mathbb {SL},\mathbb {TL},\varnothing \}$ such that *p* *o* *s* *t*_*α*_(*u,x*)∈*p* *o* *s* *t* *l* *i* *s* *t*_*e*_(*u,x*).

For example, Figures 1 c, d present two different reconciliations, between the gene tree in Fig. 1 a and the subdivision of a dated species tree in Fig. 1 b. Denote by *α* the reconciliation in the Fig. 1 d, then *α*(*w*)={*y*}, *α*(*u*)={*x* ^′′^,*A*}, *α*(*v*)={*x* ^′^,*B*},*α*(*a*)={*A*},*α*(*b*)={*B*},*α*(*c*)={*C*},*α*(*d*)={*D*}.

Note that, due to the subdivision, two events belonging to two different reconciliations located in different branches of *S* ^′^ may correspond to the same event in *S*. This implies that two reconciliations that are distinct according to Definition 2 in *S* ^′^ may be *equivalent* in *S*. We can thus define a *canonical* reconciliation [7] as the member of this equivalent set for which each event is located as low as possible within a branch.

For every pair (*u,x*) of a reconciliation *α*, where *x*=*α*_*i*_(*u*) for some *i*, with 1≤*i*≤|*α*(*u*)|, we denote by *α*(*u,x*) the reconciliation corresponding to the *restriction* of *α* on *G*_*u*_ such that the first element associated to *u* by this reconciliation is *x*; for the sake of simplicity, we call *α*(*u,x*) a reconciliation between *u* and *x*.

The fact that the solution space is finite when we disallow sequences of consecutive $\mathbb {TL}$ (and also $\mathbb {DL}$ events) is implied by the following remark, which is easily deduced from Definition 1:

#### **Remark1**.

If an event that is not a $\mathbb {TL}$ happens at (*u*,*α*_*i*_(*u*)), and if (*v,y*) is a pair of nodes in *p* *o* *s* *t*_*α*_(*u*,*α*_*i*_(*u*)), then either *v* is a child of *u* or *y* is a child of *α*_*i*_(*u*).

### Pareto-optimal reconciliations

Given two vectors **v**=(*d*_1_,*t*_1_,*l*_1_) and **v’**=(*d*_2_,*t*_2_,*l*_2_), we say that **v**≤**v’** if and only if *d*_1_≤*d*_2_, *t*_1_≤*t*_2_, and *l*_1_≤*l*_2_. Moreover, **v**<**v’** if and only if **v**≤**v’** and at least one entry of **v** is strictly smaller than its corresponding entry in **v’**. Denote by **v**⊕**v’** the vector (*d*_1_+*d*_2_,*t*_1_+*t*_2_,*l*_1_+*l*_2_) and by **v**⊗**v’** the value *d*_1_·*d*_2_+*t*_1_·*t*_2_+*l*_1_·*l*_2_. Note that ⊕ and ⊗ are used to denote respectively the vector addition and the dot product.

The *event count vector* (notion proposed in [10]) of an event type *e*, denoted by **v**(*e*), defines the number of duplications, transfers, and losses associated with *e*. For example, $\textbf {v}({\mathbb {TL}})= (0,1,1)$, $\textbf {v}({\mathbb {SL}})=(0,0,1)$, $\textbf {v}({\mathbb {S}})=(0,0,0)$. Let *α* be a reconciliation that contains exactly *d* duplications, *t* transfers, and *l* losses, then we denote the event count vector (*d,t,l*) of *α* as **v**(*α*). Thus, for every pair (*u,x*) of a reconciliation *α*, we denote by **v**(*α*(*u,x*)) the event count vector for *α*(*u,x*). Since *p* *o* *s* *t*_*α*_ defines a traversal for all mappings of *α*, **v**(*α*(*u,x*)) can be computed recursively as follows: let *e* be the event type associated with (*u,x*) in *α*; if $e=\mathbb {C}$ then **v**(*α*(*u,x*))=(0,0,0), otherwise $\textbf {v}(\alpha (\textit {u,x}))=\textbf {v}(e)\oplus (\bigoplus _{(\textit {v,y})\in post_{\alpha }(\textit {u,x})}\textbf {v}(\alpha (\textit {v,y}))$).

**Fig. 3:**
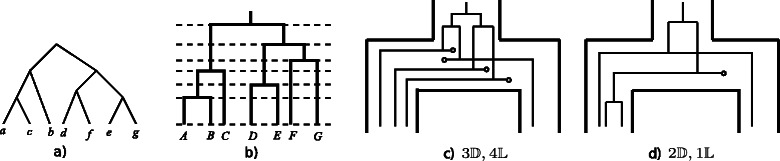
A gene tree (**a**) and species tree (**b**) for which there exists a Pareto-optimal reconciliation with 1 duplication, 2 transfers, and 4 losses that is never parsimonious for any set of positive costs. The sub optimal reconciliation *α* in (**c**) is encompassed by the reconciliation *α* ^′^ in (**d**) since *α* contains 2 duplications, 4 losses while *α* ^′^ has 2 duplications, and 1 loss

A reconciliation *α* is said to be *Pareto-optimal* if and only if there is no reconciliation *α* ^′^ such that **v**(*α* ^′^)<**v**(*α*). For example, the reconciliation in Fig. 3 c is not Pareto-optimal because there exists a reconciliation with a smaller event count vector (e.g. Fig. 3 d).

### Parsimonious reconciliation with respect to a cost range

Let *δ*,*τ*,*λ* be three positive real numbers that correspond respectively to the costs for a $\mathbb {D}$, a $\mathbb {T}$, and a $\mathbb {L}$ event. For every pair (*u,x*) of a reconciliation *α*, the cost of *α*(*u,x*) with respect to the cost vector **c**=(*δ*,*τ*,*λ*), denoted by *c* *o* *s* *t*(*α*(*u,x*),**c**), is defined by **v**(*α*(*u,x*))⊗**c**. Hence, the cost of *α* with respect to **c** is *c* *o* *s* *t*(*α*,**c**)=**v**(*α*)⊗**c**. A reconciliation *α* is said to be *parsimonious* with respect to a cost vector **c** if and only if there is no reconciliation *α* ^′^ such that *c* *o* *s* *t*(*α* ^′^,**c**)<*c* *o* *s* *t*(*α*,**c**).

#### **Remark2**.

If a reconciliation *α* is parsimonious with respect to a certain cost vector (*δ*,*τ*,*λ*), then it must be Pareto-optimal. However, the converse is not ensured if only strictly positive costs are considered. For example, there exist five Pareto-optimal reconciliations for the gene tree in Fig. 3 a and the species in Fig. 3 b, associated with the following event count vectors: *v*_1_=(0,4,0),*v*_2_=(0,3,2),*v*_3_=(1,1,5),*v*_4_=(1,2,4) and *v*_5_=(2,0,7). The reconciliation associated with *v*_4_ is Pareto-optimal but never parsimonious for any set of positive costs. Indeed, if *τ*≤*λ*, then the solution (0,4,0) is more parsimonious than (1,2,4), because 4*τ*<*δ*+6*τ*≤*δ*+2*τ*+4*λ*. If *τ*>*λ*, then the solution (1,1,5) is more parsimonious than (1,2,4) because *δ*+*τ*+5*λ*<*δ*+2*τ*+4*λ*.

Therefore, the space of all Pareto-optimal reconciliations is not necessarily equivalent to the one of all parsimonious reconciliations.

Since the cost is unit-less, and the solutions depend on the ratios between the event costs rather than on the absolute costs, we define, for each cost vector **c**=(*δ*,*τ*,*λ*), the ratio cost vector associated with **c**, denoted by **r**(**c**), as (*λ*/*τ*,*δ*/*τ*,*λ*/*δ*). Given two vectors **r**_*m*_ and **r**_*M*_, a reconciliation *α* is said to be *parsimonious with respect to the ratio cost range* [**r**_*m*_;**r**_*M*_] if and only if there exists at least one cost vector **c** such that **r**_*m*_≤**r**(**c**)≤**r**_*M*_, and *α* is parsimonious with respect to **c**. A first straightforward formulation of the suboptimal reconciliation problem can be stated as follow. **Problem 1:** All Parsimonious Reconciliations With Respect to a Ratio Cost Range. **Input:** A dated species tree *S*, a gene tree *G* such that $\mathcal {L}(G) \subseteq \mathcal {L}(S)$, two ratio cost vectors **r**_*m*_,**r**_*M*_. **Output:** The set $\mathcal {R}_{o}$ of all parsimonious reconciliations between *G* and *S* ^′^ with respect to the cost range [**r**_*m*_;**r**_*M*_].

Note that the set $\mathcal {R}_{o}$ can have an exponential size but can be represented in a polynomial-size graph, as we shall see soon.

### *ε*-Pareto-optimal reconciliations

A given cost vector may fail to yield to the true gene evolution; this happens when the latter is not parsimonious with respect to the given cost vector. This is why we extend here the concepts presented previously in this section to suboptimal reconciliations. Given a dated species tree *S*, a gene tree *G* and a cost vector **c**, denote by *c* *o* *s* *t* ^*m*^(*G,S* ^′^,**c**) the cost of a parsimonious reconciliation between the subdivision *S* ^′^ of *S* and *G* with respect to **c** (this value can be computed in *O*(|*V*(*S*)|^2^·|*V*(*G*)|) time [1]). Then, given an over-cost *ε*, we will consider all reconciliations that have cost at most *c* *o* *s* *t* ^*m*^(*G,S* ^′^,**c**)+*ε*. However, allowing a tolerance in the cost can lead to reconciliation sets containing pairs of reconciliations *α*,*α* ^′^ such that *α*≠*α* ^′^ and **v**(*α* ^′^)<**v**(*α*). To avoid this, we consider reconciliations with cost at most *c* *o* *s* *t* ^*m*^(*G,S* ^′^,**c**)+*ε* only if they are Pareto-optimal, and we call them *ε*-Pareto-optimal reconciliations. Moreover, note that by allowing an over-cost *ε*, we not only consider the reconciliations that are suboptimal with respect to the input cost vector, but also consider some reconciliations that are parsimonious with respect to some other cost vectors. Hence, this approach allows us to implicitly vary the input costs.

The second problem that we are interested in here is the following: **Problem 2:** All *ε*-Pareto-optimal Reconciliations **Input:** A dated species tree *S*, a gene tree *G* such that $\mathcal {L}(G) \subseteq \mathcal {L}(S)$, a cost vector **c**=(*δ*,*τ*,*λ*) for $\mathbb {D}$, $\mathbb {T}$ and $\mathbb {L}$ events, and an over-cost *ε*. **Output:** The set $\mathcal {R}_{\textit {so}}$ of all reconciliations *α* between *G* and *S* ^′^ such that *c* *o* *s* *t*(*α*,**c**)≤*c* *o* *s* *t* ^*m*^(*G,S* ^′^,**c**)+*ε* and *α* is Pareto-optimal.

## Methods

In this section, we will show how to compute all event count vectors of Pareto-optimal reconciliations (see Algorithm 1). This list of vectors is cleaned to keep either reconciliations that are parsimonious with respect to the given cost range (for solving Problem 1), or those having cost at most *c* *o* *s* *t* ^*m*^(*G,S* ^′^,**c**)+*ε* (for solving Problem 2). Each of the returned lists is then used to construct the corresponding graph (Algorithm S1 in the Additional file 1) that represents all the solutions for the associated problem. Using this graph, we can compute supports for events, as well as median reconciliations.

### Computing all event count vectors of parsimonious reconciliations for a given cost range (Algorithm 1)

Here, we adapt the algorithm from [10] to the case of dated species tree. We start by describing the two operations ⊕_*p*_ and *concatPareto* used in Algorithm 1.

A list *L* of vectors is said to be *Pareto-optimal* if and only if, for every vector **v** in *L*, there is no vector **v’** in *L* such that **v’**≤**v**. Given two lists of vectors *L*_1_, and *L*_2_, denote by *L*_1_⊕_*p*_ *L*_2_ the list consisting of all Pareto-optimal vectors **v** such that there exists at least a vector **v**_*i*_ in *L*_*i*_ for every *i*∈{1,2} such that **v**=**v**_1_⊕**v**_2_. The method *c* *o* *n* *c* *a* *t* *P* *a* *r* *e* *t* *o*(*L*_1_,*L*_2_) concatenates the lists *L*_1_ and *L*_2_ and ensures that the resulting list is a Pareto-optimal one by removing its non Pareto elements. The implementation of these two operations are detailed in the Additional file 1 (see proof of Theorem 1).

For all *u*∈*V*(*G*) and *x*∈*V*(*S* ^′^), denote by $\mathcal {C}(\textit {u,x})$ the list consisting of all triplets (*d,t,l*) such that there exists at least a Pareto-optimal reconciliation between *u* and *x* that has (*d,t,l*) as its event count vector. Algorithm 1 starts by computing $\mathcal {C}(\textit {u,x})$ for all *u*∈*V*(*G*) and *x*∈*V*(*S* ^′^) (lines 3–19). Note that, from Definition 2 and Remark 1, the list $\mathcal {C}(\textit {u,x})$ can be computed recursively from those of the mappings in *p* *o* *s* *t* *l* *i* *s* *t*_*e*_(*u,x*) for all event types except for $e=\mathbb {TL}$ in a bottom-up order of *V*(*G*) and increasing time order of *V*(*S* ^′^). This is done on lines 6–16 of Algorithm 1. However, since consecutive $\mathbb {TL}$ events are not allowed, the event count vectors due to $\mathbb {TL}$ can be computed once those of all other events have been computed for all nodes in the same time slice as *x* (line 19), via the function *bestTriplets* (more on this below). Indeed, $\mathbb {TL}$ events are defined only for nodes of *S* ^′^ in the same time slice. The event count vectors of all Pareto-optimal reconciliations between *G* and *S* ^′^, denoted by *P* *O*(*G,S* ^′^), are thus those in $concatPareto_{x\in V(S')}(\mathcal {C}(r(G),x))$ (line 20). The operation *computePars* (line 21) filters from the latter list the reconciliations that are not parsimonious with respect to the input cost range. This filtering is done in the same way as in [10] by normalizing the cost vector (e.g. fixing *τ*=1). Then a vector *v*∈*P* *O*(*G,S* ^′^) is retained if and only if the linear system of inequalities where **v**⊗(*δ*,1,*λ*)≤**v’**⊗(*δ*,1,*λ*) for every **v’**∈*P* *O*(*G,S* ^′^) and **v’**≠**v** has at least one solution (*λ*,*δ*) such that **r**_*m*_≤(*λ*,*δ*,*λ*/*δ*)≤**r**_*M*_. Following [10], this can be done in time *O*(|*V*(*G*)|^2^·*l* *o* *g*(|*V*(*G*)|)) for each vector **v**, and *O*(|*V*(*G*)|^4^·*l* *o* *g*(|*V*(*G*)|)) for all the list *P* *O*(*G,S* ^′^).



Note that, in Algorithm 1, to speed up the computation of transfer events, we use the fact that the event count vectors of the receivers must also form a Pareto-optimal list (this observation can be trivially proved). Hence, we compute for each mapping (*u,x*) the list *b* *e* *s* *t* *T* *r* *i* *p* *l* *e* *t* *s*(*u,x*), which is the Pareto-optimal vector list containing all vectors **v** such that there exists at least a node *z*≠*x* (since the receiver must be different from the donor) at the same time slice as *x* and $\textbf {v} \in \mathcal {C}(\textit {u,z})$. Again to speed up the algorithm, we can compute, for each time slice *ts*, the Pareto-optimal list *b* *e* *s* *t* *T* *r* *i* *p* *l* *e* *t* *s*(*u,ts*) which consists of all vectors **v** such that there exists at least a node *z*∈*V*_*ts*_(*S* ^′^) and $\textbf {v}\in \mathcal {C}(\textit {u,z})$. Then, *b* *e* *s* *t* *T* *r* *i* *p* *l* *e* *t* *s*(*u,x*) for all *x*∈*V*_*ts*_(*S* ^′^) can be deduced from *b* *e* *s* *t* *T* *r* *i* *p* *l* *e* *t* *s*(*u,ts*) by removing from it all triplets that are only contained in $\mathcal {C}(\textit {u,x})$.

#### **Theorem1**.

Algorithm 1 returns a matrix $\mathcal {C}$ such that a reconciliation *α* between *G* and *S* ^′^ is parsimonious with respect to the range [**r**_*m*_,**r**_*M*_] if and only if $\mathcal {C}(r(G),\alpha _{1}(r(G)))$ contains **v**(*α*). The complexity of Algorithm 1 is *O*(|*V*(*S*)|^2^×|*V*(*G*)|^5^), and the algorithm can be implemented in a space complexity of *O*(|*V*(*S*)|^2^×|*V*(*G*)|^3^).

The algorithm is very similar to the algorithm presented in [10], except that: 1) we process the operation ⊕_*p*_ for two lists of size *k* in times *O*(*k* ^2^) instead of *O*(*k* ^2^ *l* *o* *g*(*k*)) [10] by using sorted lists; 2) we consider the dated version of the reconciliation problem while in [10] they consider the undated one; 3) we take into account $\mathbb {TL}$ events, which are not considered in the model of [10].

The proof of Theorem 1 is deferred to the Additional file 1.

### Computing all event count vectors of *ε*-Pareto-optimal reconciliations

Problem 2 can be solved similarly to Problem 1 as shown in the following lemma, whose proof is deferred to the Additional file 1.

#### **Lemma1**.

Let *α* be a reconciliation between *G* and *S* ^′^. If *α*(*u,x*) is an *ε*-Pareto-optimal reconciliation between *u* and *x*, then *α*(*v,y*) is also an *ε*-Pareto-optimal between *v* and *y* for every (*v,y*)∈*p* *o* *s* *t*_*α*_(*u,x*).

For each mapping (*u,x*), denote by *c* *o* *s* *t* ^*m*^(*u,x,* **c**) the minimum cost over all reconciliations between *u* and *x* with respect to the cost vector **c**. By Lemma 1, the set of all event count vectors for all *ε*-Pareto-optimal reconciliations can be computed by Algorithm 1 with a small modification: for each pair (*u,x*) only event count vectors with an associated cost that is at most *c* *o* *s* *t* ^*m*^(*u,x,* **c**) + *ε* are retained. Hence, the time complexity of this problem is at most the one of Algorithm 1.

### Representing a space of reconciliations in a compact way

There exist cases for which the number of equally optimal reconciliations is exponential with respect to the input trees size. However, by factorizing their common mappings, it is possible to store in polynomial space all optimum reconciliations within a single graph, via the reconciliation graph (or DTL-graph) [7]. This graph is a bipartite graph made of event nodes and mapping nodes. Rather than having only one node for each mapping (*u,x*), we extend this representation by associating each mapping node with an event count vector. This allows us to keep track of the different combinations of event counts a mapping (*u,x*) may be associated to in different reconciliations of our solution space. In more detail:

#### **Definition3** (**Reconciliation graph (adapted from [**7**])**).

A reconciliation graph $\mathcal {G}$ for *G* and *S* ^′^ is an acyclic directed bipartite graph that contains two kinds of nodes – event nodes and mapping nodes. An event node is associated with an event type $\mathbb {C}$, $\mathbb {S}$, $\mathbb {D}, \mathbb {T}, \varnothing, \mathbb {SL}$, or $\mathbb {TL}$. A mapping node *m* is associated with (*u,x,* **v**) where *u*∈*V*(*G*), *x*∈*V*(*S* ^′^), and $\textbf {v}=(\textit {d,t,l})\in \mathbb {N}^{3}$; we denote *u* as *m*_*G*_, *x* as ${{m}_{S'}\phantom {\dot {i}\!}}$, and **v** as *m*_**v**_. The following properties hold:

1. The root set of $\mathcal {G}$ consists of mapping nodes *m* where *m*_*G*_=*r*(*G*);
2. For every mapping node *m*=(*u,x,* **v**), if *u*∈*L*(*G*), *x*∈*L*(*S* ^′^) and *s*(*u*)=*s*(*x*), then **v**=(0,0,0), and *m* has one unique child of type $\mathbb {C}$. Otherwise, *m* has a non-empty set of children and each one has a type different than $\mathbb {C}$;
3. For every event node *n*_*e*_ of $\mathcal {G}$ that is associated with type *e*, *n*_*e*_ has one unique parent, which is a mapping node (*u* ^′^,*x* ^′^,**v’**). If $e=\mathbb {C}$, then *n*_*e*_ is a leaf of $\mathcal {G}$. Otherwise, the children set of *n*_*e*_ is not empty and: a) either $e\in \{\mathbb {S},\mathbb {D},\mathbb {T}\}$, and *n*_*e*_ has two children *m*_1_,*m*_2_ such that {*m*_1_,*m*_2_}∈*p* *o* *s* *t* *l* *i* *s* *t*_*e*_(*u* ^′^,*x* ^′^); or $e\in \{\mathbb {SL},\mathbb {TL},\varnothing \}$ and *n*_*e*_ has only one child *m*_1_ such that *m*_1_∈*p* *o* *s* *t* *l* *i* *s* *t*_*e*_(*u* ^′^,*x* ^′^), b) $\textbf {v'} =\textbf {v}(e)\oplus (\bigoplus _{m\in children(n_{e})} m_{\textbf {v}})$.

**Fig. 4:**
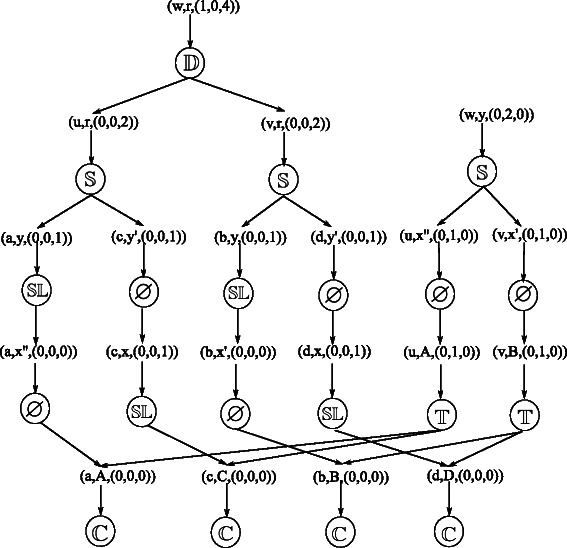
The reconciliation graph that represents the two reconciliations in Fig. 1 **c**, **d**. Circles indicate event nodes – the event type is specified inside the circle. The remaining nodes are mapping ones and the vectors associated to them specify respectively the node of the gene tree, the node of the species tree, and the associated number of duplications, transfers, and losses

For example, Fig. 4 presents the reconciliation graph that contains the two reconciliations depicted in Fig. 1 c, d. In this example, each mapping node has one child and at most one parent, but in general, each mapping node can have several children and several parents.

#### **Definition4** (**Reconciliation tree (adapted from [**7**])**).

A *reconciliation tree* of *G* and *S* ^′^ is a reconciliation graph of *G* and *S* ^′^ that has one root, and each mapping node has precisely one child. A reconciliation tree *T* of *G* and *S* ^′^ *depicts* a reconciliation *α* of *G* and *S* ^′^ if and only if the root of *T* has form (*r*(*G*),*α*_1_(*r*_*G*_),**v**(*α*)), and for each mapping (*u,x*) of *α*, there exists one and only one mapping node (*u,x,* **v**) of *T* where **v**=**v**(*α*(*u,x*)).

Note that the definition of reconciliation tree given in [7] is actually the same as the one given in Definition 4, but here we reformulated it to be in agreement with the new definition of a reconciliation graph. Following [7], each reconciliation tree *depicts* a unique reconciliation and conversely each reconciliation is depicted by one reconciliation tree.

#### **Definition5** (**Full subtree**).

Let $\mathcal {G}$ be a reconciliation graph of *G* and *S* ^′^, a connected subtree *T* of $\mathcal {G}$ is a full subtree of $\mathcal {G}$ if and only if:

- the root of *T* is a mapping node *m* where *m*_*G*_=*r*(*G*);
- each mapping node of *T* has precisely one child;
- each event node of *T* has the same children set in *T* as in $\mathcal {G}$;
- all leaves of *T* are leaves in $\mathcal {G}$.

For example, in Fig. 4 the graph has two roots. Starting from one root, and going down to the leaves, we can obtain a full subtree of this graph. Here, each mapping node has only one child, so there are only two full subtrees. In general, each root of the graph can correspond to several full subtrees.

#### **Lemma2**.

Given a reconciliation graph $\mathcal {G}$ of *G* and *S* ^′^, every full subtree *T* of $\mathcal {G}$ is a reconciliation tree of *G* and *S* ^′^, i.e. *T* depicts a reconciliation between *G* and *S* ^′^.

#### *Proof*.

Since *T* is a connected subtree of $\mathcal {G}$ and *T* has the four properties in Definition 5, it is easy to check that *T* respects the three conditions required to be a reconciliation graph according to Definition 3. Moreover, *T* has a unique root and each of its mapping node has a unique child, thus it respects the definition of a reconciliation tree (Definition 4).

Given a matrix of event count vectors, its reconciliation graph can be constructed by a backtracking process, that searches, for each event count vector, all possible events associated to it that can occur in a reconciliation. Moreover, each mapping node appears just once in the graph. This process is described by Algorithm S1 in the Additional file 1.

#### **Theorem2**.

Algorithm S1 runs in *O*(|*V*(*S*)|^3^×|*V*(*G*)|^5^) space and time complexity. The returned graph $\mathcal {G}$ is a reconciliation graph of *G* and *S* ^′^ such that, every full subtree of $\mathcal {G}$ depicts a reconciliation between *G* and *S* ^′^ whose event count vector is contained in $\mathcal {C}$; and conversely, every reconciliation between *G* and *S* ^′^ whose event count vector is contained in $\mathcal {C}$ is depicted by a full subtree of $\mathcal {G}$. Moreover, $\mathcal {G}$ has the minimum number of vertices among the graphs having this property.

The proof of Theorem 2 is deferred to the Additional file 1.

### Support and median reconciliation

Recall that in this paper we are interested only in canonical reconciliations [7]. Thus, for the sake of simplicity, hereafter reconciliations will always be assumed to be canonical.

The *support* of an event *e*, denoted by *f*(*e*), is defined as the percentage of reconciliations containing this event, i.e. its frequency in the space of considered reconciliations $\mathcal {R}$. Once the graph $\mathcal {G}$ has been computed (Algorithm S1 in the Additional file 1), we can compute the frequency *f*(*e*) associated with any event *e* within the set of reconciliations represented by $\mathcal {G}$ via two traversals of this graph. The outline of the algorithm is similar to the one given in [7]. First, via a post-order traversal of $\mathcal {G}$, we compute, for each node *z* of the graph, the value *s* *c* *o* *r* *e*(*z*) as described in Algorithm 5 of [7]. The value *s* *c* *o* *r* *e*(*z*) corresponds to the number of possible subtrees of the graph $\mathcal {G}$ rooted at *z* that are contained in a full subtree of $\mathcal {G}$. Second, using a pre-order traversal, we can compute, for each node *z*, the value *r* *e* *c* *N* *u* *m*(*z*), which corresponds to the number of reconciliations between *G* and *S* ^′^ that contain *z*, using the following recursive property. If *m*_*G*_(*z*)=*r*(*G*), then *r* *e* *c* *N* *u* *m*(*z*)=*s* *c* *o* *r* *e*(*z*). Otherwise, if *z* is a mapping node that has *p*_1_,…,*p*_*k*_ as parents (all are event nodes), then $recNum(z)=\sum _{i=1}^{k} recNum(p_{i})$. If *z* is an event node that has *p* as its parent, then *r* *e* *c* *N* *u* *m*(*z*)=*r* *e* *c* *N* *u* *m*(*p*)·*s* *c* *o* *r* *e*(*z*)/*s* *c* *o* *r* *e*(*p*). Since in our generalization, each mapping can be associated with different event count vectors, then each event may be present in more than one event node. The frequency of each event *e* is thus obtained by summing the frequencies *f*(*n*_*e*_) of all event nodes *n*_*e*_ that represent this specific event.

Dealing with a set of reconciliations can be cumbersome, especially for further analysis and biological interpretations where a single reconciliation will suffice. Rather than randomly picking a reconciliation among the parsimonious ones, in [8] the authors proposed to return one minimizing the sum of event-based distances between itself and all other parsimonious alternatives. The authors also proved that this so called *median reconciliation* can be computed by two traversals of $\mathcal {G}$. Their algorithm can easily be adapted to our generalization of the reconciliation graph. Hence, we consider the median reconciliation of $\mathcal {G}$ as the output reconciliation of our methods, using as event-based (symmetric) distance between two reconciliations the number of events present in only one of the two reconciliations (as done in [8]).

## Experiments

### Simulated data and compared methods

Experiments were conducted on the simulated data set in [8, 12], available at http://www.atgc-montpellier.fr/Mowgli/, which we briefly describe below. To construct the data set, first 1000 evolutionary histories, composed of $\mathbb {D}, \mathbb {T}, \mathbb {L}$ and $\mathbb {S}$ events, were simulated according to a birth and death process along a phylogeny of 37 proteobacteria [13]. Rates for macro-evolutionary events are as follows: (a) the loss rate was randomly chosen in the [ 0.001,0.0018] interval, where the units are events per gene per million years. Moreover, the ratio between the birth rate (sum of the duplication and transfer rates) and the loss rate was randomly chosen in the [ 0.5,1.1] interval, while the proportion of the duplication rate to the birth rate was randomly chosen in the [ 0.7,1] interval. This led to 1000 simulated gene trees (*G*_*True*_) on which the numbers of speciations, duplications, transfers, and losses in average are respectively 29.75, 3.87, 0.88, and 8.26. The mean number of genes per family in this dataset is 28.9, the largest family has 67 genes and the smallest 11 genes. Each family is found in 20.88 species in average, with a minimum of 5 and a maximum of 33 species. These gene trees were used to generate DNA sequences with the Seq-Gen program [14]. From these sequences, RAxML [15] was used to infer 1000 maximum likelihood gene trees (*G*_*ML*_). For more details, we refer to [8], [12] Results section.

We used the so-obtained 1000 pairs of dated species tree/gene trees to compare the performances of the two approaches presented in this paper (strategies s3–s5) with the ones presented in [7, 8] and [10] (strategies s1, s2, s6 and s7 respectively, more details below). We did not test the method presented in [9] as the software is not yet available.

For testing the method in [10], we used the tool evenscape of the software Xscape (version used in [10]). Note that Xscape considers the species tree as undated. Several options to compute event supports are proposed by this program; we tested them all, and present here the ones that give the best accuracy: supports computed using option ‘I’, based on the number of regions, without taking into account the area of the regions. A list of the options and their description is given in the Additional file 1.

Meaningful cost vectors can be obtained from the real simulated frequencies of each event type as described in [12, see Equation 1 of the *Experiments on simulated datasets* section]: if a real gene history encompasses *n*_*tot*_ events (duplications, transfers and losses) among which *n*_*e*_ are duplications (respectively transfers and losses), then the cost of a duplication (respectively of a transfers and of a loss) is *l* *o* *g*(*n*_*tot*_/*n*_*e*_). This cost vector has been already used in the literature, for example to test a method to correct gene trees via reconciliations [12], and to test the sample-based method for computing event supports [8] on the same data set. Hence, we use it here for our experiments. Since, in real applications, these frequencies are unknown, we tested a second cost vector, which is a standard cost vector (2,3,1) for duplications, transfers and losses that is used in several studies [13; 16, among others]. Moreover, for the region-based method, the standard cost range used in [10] was also tested (strategy 6). Hence, we have tested the following methods for each cost vector (except strategy 6 that always uses the cost range given in [10]). Other cost vectors could be proposed and tested, but this is beyond the scope of this paper.

**MPR one cost**: This strategy computes the median reconciliation, as well as the event supports, via the optimal reconciliation graph, that is the graph containing all parsimonious reconciliations for the given cost vector [7].
**MPR sampled costs**: This strategy is the one presented in [8]. For each gene tree $G^{i}_{\textit {ML}}$, we generated a set of 1000 cost vectors around the initial cost of $G^{i}_{\textit {ML}}$ using a noise level of 20 *%* for cost vector 1, and 5 *%* for cost vector 2 – these are the noise levels that gave the best results among the ones we tested on our simulated data. The 1000 optimal reconciliation graphs constructed for each sampled cost vector are then combined in a unique graph that is used to compute the event supports and to construct the median reconciliation.
**MPR cost range**: This strategy corresponds to Problem 1. We computed the graph containing all the parsimonious reconciliations with respect to a cost range, which are used to compute the median reconciliation as well as the event supports. The input ratio cost range [**r**_*m*_,**r**_*M*_] was chosen around the ratio **r**(**c**)=(*λ*/*τ*,*δ*/*τ*,*λ*/*δ*) of the input cost **c** by varying the two last ratios (*δ*/*τ* and *λ*/*δ*) of ±80 *%* for cost vector 1, and ±40 *%* for cost vector 2. We did not constrain the first ratio cost (*λ*/*τ*) and let this one be defined by the two others (we did so because the method in [10] does not restrict this ratio, and we want to compare the two methods on the same ranges. Nevertheless, our method can restrict this ratio and, when we do so, we observe a slight improvement – results not shown). For example, for cost vector 2, the initial ratio is (1/3,2/3,1/2), which gives us the ranges [ 60 *%*·2/3;140 *%*·2/3] and [ 60 *%*·1/2;140 *%*·1/2] for the two last ratios, implying that the range for the first ratio is [ 60 *%*·60 *%*·1/3;140 *%*·140 *%*·1/3]. Hence, we have **r**_*m*_=(0.12,0.4,0.3) and **r**_*M*_=(0.653,0.933,0.7).
***ε*** **-Pareto**: This strategy corresponds to Problem 2. It computes the median reconciliation – as well as the event supports – via the *ε*-Pareto optimal reconciliation graph, which is the graph containing all *ε*-Pareto optimal reconciliations. This strategy differs from the first one by the set of reconciliations it takes into account to construct the reconciliation graph. The choice of *ε* is described below.
***ε*** **-Pareto** ^**∗**^: Similar to strategy s4, but we added more constraints on the retained event count vectors such that, for every retained event count (*d,t,l*), it does not exist any (*d* ^′^,*t* ^′^,*l* ^′^) with *l* ^′^≤*l*, *t* ^′^≤*t*, and *d* ^′^+*t* ^′^≤*d*+*t* (see the Discussion Section for an explanation on why this latter constraint was added). Note that other types of constraints can be easily integrated into this model.
**Region-based**: This strategy uses the tool *eventscape* of *Xscape*, which is an implementation of the method described in [10]. Since *Xscape* does not generate reconciliations, this software is used to assign supports to our best median reconciliations – namely the ones of strategy s3 with cost vector (2,3,1). We do this instead of using the full list of events returned by Xscape because the full list provides too many events leading to a large number of false positives; moreover, many events in the list cannot happen in a same reconciliation. This choice gives indeed substantially better results than using the Xscape list (data not shown). The input cost range was chosen as the one used in [10]: *λ*/*δ* in [ 0.1;5], and *τ*/*δ* in [ 0.1;5]. Hence,we have **r**_*m*_=(0.02,0.2,0.1) and **r**_*M*_=(50,10,5).
**Region-based (small range)**: Similar to the previous strategy, but we used the same input cost ranges as strategy s3. Note that these ranges are smaller than the one used in the strategy 6. As for strategy s6, the event lists are obtained from the median reconciliations of strategy s3.

For the strategies that use the *ε*-Pareto optimality, the over-cost *ε* was chosen as the difference between the input transfer cost and duplication cost. This choice is based on the fact that the topological differences between a species tree and a gene tree can be explained by either duplications or transfer events. This over-cost permits some duplications to change into transfer events (and vice versa) if the resulting reconciliations are not too far (that is, their over-cost is smaller than *ε*) from the parsimonious ones. Obviously, there can be other adequate choices for the over-cost, and depending on the data, one should choose an appropriate over-cost.

The first five strategies all use dated species trees, reconciliation-based supports, and generate median reconciliations. The two last strategies constitute a second group that uses undated species trees, region-based supports, and does not generate median reconciliations.

For each strategy *s*_*j*_ with 1≤*j*≤5, denote by $\hat {\mathbb {E}}^{i}_{s_{j}}(t)$ the set of all events obtained from the median reconciliation given by strategy *s*_*j*_ on the gene tree $G^{i}_{\textit {ML}}$ that have supports above a given threshold *t*. For s6 and s7, $\hat {\mathbb {E}}^{i}_{s_{j}}(t)$ has the same signification, but, as already mentioned, the supports are calculated by the strategy while the sets of events are those of the median reconciliations computed by strategy s3 with cost vector (2,3,1).

## Results and discussion

As was done in previous papers [1, 8, 12], the reconciliation error for each strategy *s*_*j*_, gene tree $G^{i}_{\textit {ML}}$, and threshold *t* was measured on $\mathbb {D}$, $\mathbb {T}$ and $\mathbb {L}$ events using the symmetric distance between the set of predicted events ($\hat {\mathbb {E}}^{i}_{s_{j}}(t)$) and the true event set ($\mathbb {E}^{i}_{\textit {True}}$):

$d\left (\hat {\mathbb {E}}^{i}_{s_{j}}(t),\mathbb {E}_{\textit {True}}\right) = \left | \hat {\mathbb {E}}^{i}_{s_{j}}(t) \backslash \mathbb {E}_{\textit {True}}\right |_{\mathbb {D}\mathbb {T}\mathbb {L}} + \left | \mathbb {E}_{\textit {True}}\backslash \hat {\mathbb {E}}^{i}_{s_{j}}(t)\right |_{\mathbb {D}\mathbb {T}\mathbb {L}}$, where the first and second term respectively correspond to false positive ($FP^{i}_{s_{j}}(t)$) and false negative ($FN^{i}_{s_{j}}(t)$). Then, $FP_{s_{j}}(t)$ and $FN_{s_{j}}(t)$ are respectively the mean of $FP^{i}_{s_{j}}(t)$ and $FN^{i}_{s_{j}}(t)$ on all 1000 gene trees of *G*_*ML*_.

**Fig. 5:**
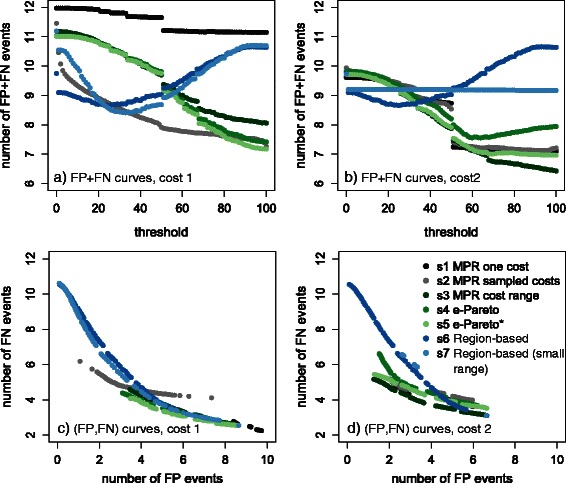
False Positives (*FP*) and False Negatives (*FN*) for the 7 strategies presented in the Experiments section on the two cost vectors described in the main text, where (**a**), (**c**) correspond to cost vector 1, while (**b**), (**d**) correspond to cost vector 2. For each strategy *s*_*j*_, the associated curve in (**a**), (**b**) consists of the points $(t,FP_{s_{j}}(t)+FN_{s_{j}}(t))$, while the associated curve in (**c**), (**d**) consists of the points $(FP_{s_{j}}(t),FN_{s_{j}}(t))$, where *t* is a threshold varying from 0–100 %, and $FP_{s_{j}}(t)$, $FN_{s_{j}}(t)$ are respectively the false positives and false negatives of strategy *s*_*j*_ after removing all events having supports smaller than *t*. Note that in Figure (**c**), (**d**), the same number of FP does not correspond to the same threshold

Figure 5 a, b show the total error rate $FP_{s_{j}}(t)+FN_{s_{j}}(t)$ of each strategy *s*_*j*_ for various thresholds, respectively for cost vectors 1 and 2. Each of the displayed curves connects the dots $(t,FP_{s_{j}}(t)+FN_{s_{j}}(t))$ for each threshold *t*=0,*t*=1,…,100. Hence, the lower the curve, the more accurate the corresponding method is. Statistical tests, mentioned in this section when comparing two strategies, have been performed using a paired t-test on 1,000 trees with a threshold p-value of 5 %, using – unless otherwise stated – the best threshold for each strategies.

Surprisingly, the first cost vector (vector 1) – although computed from the real event frequencies – is less effective for recovering the events than the default cost vector (vector 2). This is probably due to the fact that transfer events are convenient to handle gene tree reconstruction errors. Indeed, in vector 1, transfers have a very high cost since transfers are rare in the simulated gene histories. Thus, using this cost vector, the erroneous misplacement of a leaf in a gene tree, e.g. due to methodological artifacts such as long branch attraction, is preferentially explained using multiple duplication and loss events – rather than a single transfer event, as done when using vector 2 – hence inducing multiple false positive ev ents instead of a single one.

The impact of using cost vector 1 or 2 varies from one strategy to another. The most impacted strategies is s1 – that considers only reconciliations that are parsimonious for the input vector – whereas s6 – at the other extreme, does not make use of input cost vectors. The other strategies adopt a more nuanced approach, accounting for the input cost vector without blindly trusting it. This allows them to perform much better for cost vector 1 than the extreme s1 and s6 approaches. Indeed, this cost vector is inappropriate for reconciling imperfect trees (hence fully trusting its input costs is penalizing s1) but not as much as some more extreme possible costs (hence accounting for these extreme costs is penalizing s6).

Strategy s1 performs so badly with cost vector 1 while not being the best one with cost vector 2 that it seems unreasonable to rely on it while better options (e.g. strategies s2–s5) are available.

As previously shown in [8], the sampling method (strategy s2) provides a big improvement compared to s1 with cost vector 1 (Fig. 5 a). However, it has almost no impact with cost vector 2, for which s1 gives good results (Fig. 5 b).

Strategy s6 and s7 perform poorly compared to methods s2–s5 for both cost vector 1 and 2. No matter the chosen threshold, they lead to an average *F* *P*+*F* *N* above 8.5 whereas other methods reach a significantly smaller average *F* *P*+*F* *N* for high thresholds. This can be due to several reasons. First, as already mentioned, s6 does not make use of input cost vectors and can consider non biologically-relevant cost vectors. Second, both strategies have a crude “binary" approach to compute region support: a region either supports or rejects an event and we cannot differentiate the cases, for example, where 10 % of the reconciliations in a region contain this event from cases where 95 % do. Moreover, those two methods are also penalized by being the only ones dealing with undated trees. Indeed, despite using similar cost regions, strategy s3 has significantly better results than s7: s3 reaches *F* *P*+*F* *N*<8.5 for cost vector 1 when using thresholds above 70 *%* (while *F* *P*+*F* *N* is always greater than 9 with s7) and reaches *F* *P*+*F* *N*<7 for cost vector 2 when using thresholds above 70 *%* (while *F* *P*+*F* *N* is always greater than 10 with s7). Note that at threshold 100 *%*, only events of the median reconciliation that are present in all reconciliations are retained. Hence, for this extreme threshold, the only remaining difference between s3 and the less accurate strategy s7 is that s7 uses undated species trees, while s3 uses dated ones. This confirms that using dated (or at least partially dated) species trees has a strong impact on reconciliation accuracy and should be favored whenever possible.

Strategy s2 uses cost vectors directly sampled from the input one. Its results are thus more influenced by the quality of the input costs than those of the new strategies proposed in this paper, namely s3, s4 and s5. The three new strategies are thus more robust to the choice of the input cost vector than s1 and s2 while avoiding the pitfall of ignoring it as done by s6 and, to a lesser extend by s7. Strategy s4 performs better than s3 and is only slightly less accurate than s5 for cost vector 1; but the performance of s4 (relative to s3 and s5) drops for cost vector 2 – where the transfer cost is much lower. This happens because *ε*-Pareto optimal reconciliations, considered by s4, may include improbable evolutionary scenarios chaining several transfer events (for example a gene that is transferred and comes back to the donor via a $\mathbb {TL}$ events) that cannot be parsimonious under any cost vector. The constraint added to s4 to obtain strategy s5 removes all these aberrant event count vectors/reconciliations. This additional constraint indeed improves the accuracy for both input cost vectors, and more clearly for cost vector 2, where s5 significantly outperforms s4 for all thresholds.

It is not our aim to draw strong conclusions concerning the relative performance of the two cost vectors used here. What we want to point out here is the following:

- when using a single cost vector, the predicted events strongly depend on its quality, and there is currently no way to identify the best cost vector (see strategy s1 and, to a lesser extend, strategy s2);
- the extreme Pareto-only approaches may consider some scenarios that are optimal under unrealistic assumptions such as transfers being 10000 times more likely than duplications;
- the in-between solutions considering Pareto solutions that are optimal for a reasonable cost range give better results;
- event support measures based on the frequency of a event in the reconciliation space seem to work better that those based on the frequency in the cost region space.

Figure 5 c, d show the ratio between FP and FN of each method. Thresholds are not reported in the figures, but we know implicitly that the right extremity of each curve corresponds to threshold 0 while the left one corresponds to threshold 100. This is because the higher the threshold, the fewer events are retained, thus leading to fewer FP and more FN. An efficient method should not increase the number of FN when decreasing the FP. Figure 5 c, d confirm that our new filtering strategies s3, s4, and s5 do not remove too many true events when increasing the threshold. For example, s3 on cost vector 2 (Fig. 5 d) decreases FP from 6.5–1.2 while FN increase only from 3.1–5.1. Besides, while all other curves have at least 1.5 FP, the curves of s6 and s7 using cost 1 extend till 0. This means that, when increasing the threshold until 100 *%*, these two methods retain almost no events, while other methods always retain some. In other words, the median reconciliation of s3 (the one that strategies s6 and s7 use) does not contain many events whose support computed by strategies s6 and s7 is nearly 1.

Further analyses were conducted for each event type. As already noted in [10], our experiments show that predicting duplications is quite easy, while predicting transfers is harder. The reader is referred to the Additional file 1: Figures S1, S2 for more details.

The running times of all strategies on a computer equipped with a 3.2 GHz Intel Core i3 processor with 8 Gb of RAM are given in Table 1. In general, our methods (s3, s4 and s5) are 500–1000 times faster than the sampling method (s2). This is because we construct only one reconciliation graph while the sampling method constructs 1000 of them. Compared to the region-based methods of [10] (s6 and s7), we are still faster (∼20 times), even though we use a dated species tree rather than an undated one as done in [10] (usually, using the dated version of a species tree increases the complexity of *O*(*V*(*S*))). Indeed, because of the reconciliation graph, we do not need to compute and store the list of events associated with each mapping as done in [10]. We can count the number of reconciliations as well as enumerating the events and compute the median reconciliation just by traversals of the graph.

**Table 1:** Average, minimum and maximum running times – given in seconds and for a computer equipped of a 3.2 GHz Intel Core i3 processor with 8 Gb of RAM – for the 7 strategies described in the Experiments Section

	Strategy 1	Strategy 2	Strategy 3	Strategy 4	Strategy 5	Strategy 6	Strategy 7
Average	0.273	305	0.541	0.291	0.331	10.11	10.5
Min	0.086	20.5	0.132	0.09	0.089	2.61	2.35
Max	0.725	3826.3	1.53	0.886	0.963	56.75	91.15

## Conclusion

In a parsimony framework, the choice of the costs for basic events may have a strong impact on the set of predicted events. In this paper, we provide a new tool dealing with this problem, which both improves the accuracy of $\mathbb {D}, \mathbb {T}, \mathbb {L}$ events predicted by parsimonious reconciliation methods and scales up to handle the larger set of gene trees used in phylogenomic studies nowadays. This work combines the complementary ideas of the methods presented in [8] and [10] into new strategies that combine their strengths: speed and reliability. To deal with the inherent uncertainty of the input cost vectors, two approaches have been proposed and tested: explicitly providing an input cost range (strategy s3), or considering non-optimal reconciliations up to a fixed over-cost (strategies s4 and s5). Our approaches and models are flexible so that one can choose the strategy that fits the data better: considering either only parsimonious reconciliations or nearly optimal ones, choosing acceptable cost ranges, the over-cost, and even providing some-user defined additional constraints to filter the event count vectors list (see strategy s5).

Our tests on simulated data demonstrate that models using dated (or partially dated) species trees seem to provide more accurate event predictions than those using undated species trees. Moreover, in our experiments, using median reconciliations is more effective than picking a random one, and better than taking the list of all events, confirming the findings of [8]. Furthermore, our approaches are faster and more accurate than both methods in [8] and [10]. Finally, this work emphasizes the benefits of using the reconciliation graph to manage the space of reconciliations efficiently. Many other tools could be developed by using this graph, for example calculating the reconciliation whose average of the supports is maximized.

This work confirms that erroneously inferred events can be, at least in part, filtered out. This filtering could probably be further improved by also taking into account reconciliations of close alternative gene tree/species tree topologies. Yet, taking such alternative reconciliations into account is challenging, as the reconciliation graph solution [7], proposed to efficiently handle alternative reconciliations, was not designed to handle reconciliations between different gene/species trees.

## Nomenclature

*α*(*u,x*): the restriction reconciliation of *α* on *G*_*u*_ that maps *u* to *x*; *α*_*ℓ*_(*u*): the last element of *α*(*u*); *α*_*i*_(*u*): the *i* ^*th*^ element of *α*(*u*);$\mathbb {C}$: Contemporary event;$\mathbb {DL}$: Duplication Loss;$\mathbb {D}$: Duplication event;$\mathbb {L}$: Loss event;$\mathbb {SL}$: Speciation Loss event;$\mathbb {S}$: Speciation event;$\mathbb {TL}$: Transfer Loss event;$\mathbb {T}$: Transfer event;$\mathcal {C}(u,x)$: the set of event count vectors of all reconciliations between *u* and *x* of the considered problem; ⊕: vector addition; ⊕_*p*_: addition of two lists of vectors, the resulting list must be Pareto-optimal; ⊗: vector dot product;$\varnothing $: No event; *c* *o* *s* *t*(*α*,**c**): the cost of *α* with respect to the cost vector **c**; *c* *o* *s* *t* ^*m*^(*u,x,* **c**): the minimum cost over all reconciliations between *u* and *x* with respect to the cost vector **c**; *f*(*e*): the support of the event *e*; *L*_*i*_: the *i* ^*th*^ element of the list *L*; *m*_*G*_: the first element of the mapping node *m*, which is a node of the gene tree *G*; *m*_**v**_: the third element of the mapping node *m*, which is an event count vector;${{m}_{S'}\phantom {\dot {i}\!}}$: the second element of the mapping node *m*, which is a node of *S* ^′^; *p* *o* *s* *t* *l* *i* *s* *t*_*e*_(*u,x*): the set of possible next mappings of (u,x) associated with the event *e*; *u*_1_: the first child of *u*; *u*_2_: the second child of u; *u*_*p*_: the parent of u; *V*_*t*_(*T*): the set of nodes of the tree *T* having time *t*; **c**: the cost vector (*δ*,*τ*,*λ*); **r**(**c**): the ratio cost vector (*λ*/*τ*,*δ*/*τ*,*λ*/*δ*) of the cost vector **c**=(*δ*,*τ*,*λ*); **r**_*M*_: the upper bound of the ratio cost vector; **r**_*m*_: the lower bound of the ratio cost vector; **v**(*e*): the event count vector of the event *e*; **v**(*α*): the event count vector of the reconciliation *α*; *c* *o* *s* *t* ^*m*^(*G,S* ^′^,**c**): the cost of the most parsimonious reconciliation between *S* ^′^ and *G* with respect to the cost vector **c**;

## Additional file

Additional file 1
**The Additional file 1 contains the proof of Theorem 1, the graph construction algorithm, the proof of Theorem 2, and some supplementary experiment descriptions and results.** (PDF 1014 kb)
