# Supplementary material for: A fast method for calculating reliable event supports in tree reconciliations via Pareto optimality
Source: BMC Bioinformatics. 2015 Nov 14;16:384. doi: 10.1186/s12859-015-0803-x (PMC4647304; doi:10.1186/s12859-015-0803-x)
Supplement: Additional file 1 — The Additional file 1 contains the proof of Theorem 1, the graph construction algorithm, the proof of Theorem 2, and some supplementary experiment descriptions and results. (PDF 1014 kb) [file 12859_2015_803_MOESM1_ESM.pdf]

S1 Supplementary Materials

S1.1 Proof of Theorem 1

Correctness After the loop on lines 5–19, for every $u \in V(G)$ and $x \in V(S')$, $\mathcal{C}(u,x)$ contains all vectors $\mathbf{v}$ such that there exists a Pareto-optimal reconciliation between u and x with event count $\mathbf{v}$. Indeed, it is obviously true for every pair of leaves u, x such that $s(u) = s(x)$, see line 7. For the other cases, for each possible event e associated with (u,x), $\mathcal{C}(u,x)$ can be computed from the entries of $\mathcal{C}$ of the mappings contained in $postlist_e(u,x)$. Due to Remark 1, this can be done recursively following a post-order of G and a bottom-up time order of S' for all events except $\mathbb{TL}$, see lines 5-16. Moreover, in a Pareto-optimal reconciliation, no sequence of $\mathbb{TL}$ events can be present, so the vectors associated with $\mathbb{TL}$ events can be computed at once for each time slice (lines 18-19). See main text for the definition of $bestTriplets$.

Since a reconciliation between G and S can map $r(G)$ to any node x of S', in order to have the Pareto-optimal list of all even count vectors between G and S', we must concatenate all $\mathcal{C}(r(G), x)$ and clean the list to be Pareto-optimal (line 20). The so obtained list $PO(G, S')$ contains all event count vector $\mathbf{v}$ such that there exists a Pareto-optimal reconciliation between G and S' with event count $\mathbf{v}$.

Note that, since any parsimonious reconciliation must be a Pareto-optimal reconciliation, every event count vector of a parsimonious reconciliation is contained in $PO(G, S')$. Finally, this list is cleaned up (line 21) to remove those that are not parsimonious w.r.t any cost vector in the input regions $[\mathbf{r}_m, \mathbf{r}_M]$ by the same method as [1]. Hence, any event count vector in the cleaned list $PO(G, S')$ corresponds to a parsimonious reconciliation between G, S' w.r.t. to the interval $[\mathbf{r}_m, \mathbf{r}_M]$ and conversely.

Complexity The operation $concatPareto$ can be implemented efficiently in $O(k)$ time by using sorted lists where k is the size of the input lists. Let us define an order for a Pareto-optimal list Q as follows: for every $\mathbf{v} = (d, t, l)$ and $\mathbf{v}' = (d', t', l')$ in Q, we say that $\mathbf{v} \bar{<} \mathbf{v}'$ if and only if: either $(d < d')$ or $(d = d'$ and $t < t')$ (the case $d = d', t = t', l < l'$ can not exist in a Pareto-optimal list). Given two ordered Pareto-optimal lists Q_1, Q_2 of size k, we can process $concatPareto(Q_1, Q_2)$ in $O(k)$ such that the resulting list is also an ordered Pareto-optimal one. It suffices to use two iterators simultaneously for the two lists, and compare each time the two current iterators to decide which one is put in the resulting list.

By using the operation $concatePareto$, the operation $\oplus_p$ can be implemented in $O(k^2)$ time if both input and output lists have size at most k. Let Q_1, Q_2 be two ordered Pareto-optimal lists that have size at most k. Denote by $\mathbf{v}_1^i$ and $\mathbf{v}_2^i$ respectively the ith vector of Q_1 and Q_2, and by Q_1^i the list consisting of the first i vectors of Q_1. Let $R_i = Q_1^i \oplus_p Q_2$, hence by hypothesis, R_i always has size at most k. Note that R_1 can be computed in $O(k)$ and that, for $k > 1$, $R_i = concatPareto(R_{i-1}, \mathbf{v}_i \oplus_p Q_2)$ can be computed in $O(k)$ when R_{i-1} is known since both R_{i-1} and $\mathbf{v}_1^i \oplus_p Q_2 = concatPareto(\mathbf{v}_1^i \oplus \mathbf{v}_2^1, \cdots, \mathbf{v}_1^i \oplus \mathbf{v}_2^k)$ are ordered Paretol-optimal lists and have size at most k. Thus, $R_k = concatPareto(R_{k-1}, \mathbf{v}_1^k \oplus_p Q_2) = Q_1 \oplus_p Q_2$ can be computed in $O(k^2)$.

Let m be the maximum size of a Parteo-optimal list where each element is an event count vector. Following [1], m is bounded by $O(|V(G)|^2)$.

Compute $bestTriplets(u, ts)$ in line 17 can be processed by using $concatPareto$ on all lists $\mathcal{C}(u, x)$ where $x \in V_{ts}(S')$. Therefore, it takes $O(|V_{ts}(S')| \cdot m)$ for each u, each time slice ts, and so $O(|V(G)| \cdot |V(S')| \cdot m)$ in total.

The block from lines 6 to 16 of Algorithm 1 requires $|V(G)| \cdot |V(S')|$ iterations. In each iteration, there are three main operations: $\oplus_p$, $concatPareto$ and $bestTriplets$. The first two operations take $O(m^2)$ time, since all lists involved in the operations (i.e. input and output ones) have size at most m. $bestTriplets(u, x)$ can be computed in $O(m)$ time from $bestTriplets(u, ts)$, that has already been computed in previous iteration at line 17. It suffices to put a label x to each triplet of $bestTriplets(u, ts)$ that is only contained in an entry $\mathcal{C}(u, x)$. Then $bestTriplet(u, x)$ consists of every triplet of $bestTriplet(u, ts)$ that is not labelled by x. Since $bestTriplet(u, ts)$ is also a Pareto-optimal list, it has size $O(m)$. Hence, each iteration takes $O(m^2)$ for each u, each x, and so $O(|V(G)| \cdot |V(S')| \cdot m^2)$ in total.

Line 19 can be processed in $O(m^2)$ for each u, each x, and so $O(|V(G)| \cdot |V(S')| \cdot m^2)$ in total. Hence, the total complexity is $O(|V(G)| \cdot |V(S')| \cdot m^2) = O(|V(G)|^5 \cdot |V(S')|)$. The space of all lists $\mathcal{C}(u, x)$ is $O(|V(G)|^3 \cdot |V(S')|)$ because there are $|V(G)| \cdot |V(S')|$ possible pairs (u, x) and each pair is associated with a list of size at most $O(|V(G)|^2)$. Since $O(|V(S')|) = O(|V(S)|^2)$, the complexity follows.

S1.2 Proof of Lemma 1

Suppose that $post_\alpha(u, x)$ contains two mappings $\{(v, y), (w, z)\}$. (The proof is similar for the case where $post_\alpha(u, x)$ consists of a single mapping). It is easy to see that $\alpha(v, y)$ is a Pareto-optimal reconciliation between v and y. It remains to prove that $cost(\alpha(v, y), \mathbf{c}) \leq cost^m(v, y, \mathbf{c}) + \epsilon$. Let e be the event associated with (u, x) in α, and let $cost(e)$ be the cost of e. By definition, we have: $cost(\alpha(u, x), \mathbf{c}) = cost(\alpha(v, y), \mathbf{c}) + cost(\alpha(w, z), \mathbf{c}) + cost(e)$.

By hypothesis, $cost(\alpha(u, x), \mathbf{c}) \leq cost^m(u, x, \mathbf{c}) + \epsilon$, and we know that $cost(\alpha(w, z), \mathbf{c}) \geq cost^m(w, z, \mathbf{c})$ because $cost^m(w, z, \mathbf{c})$ is, by definition, the minimum cost over all reconciliations between w and z. It follows that: $cost(\alpha(v, y), \mathbf{c}) = cost(\alpha(u, x), \mathbf{c}) - cost(\alpha(w, z), \mathbf{c}) - cost(e) \leq cost^m(u, x, \mathbf{c}) + \epsilon - cost^m(w, z, \mathbf{c}) - cost(e)$.

Moreover, $cost^m(u, x, \mathbf{c}) \leq cost^m(v, y, \mathbf{c}) + cost^m(w, z, \mathbf{c}) + cost(e)$ because $cost^m(u, x, \mathbf{c})$ corresponds to the minimum value among all possible events comprising e, hence: $cost(\alpha(v, y), \mathbf{c}) \leq \big(cost^m(v, y, \mathbf{c}) + cost^m(w, z, \mathbf{c}) + cost(e)\big) + \epsilon - cost^m(w, z, \mathbf{c}) - cost(e) = cost^m(v, y, \mathbf{c}) + \epsilon$.

S1.3 Graph construction algorithm

The reconciliation graph can be computed by backtracking the matrix containing event count vectors $\mathcal{C}$. This backtracking process is described in Algorithm S1, which progressively constructs the graph $\mathcal{G}$ based on a list Q of mapping nodes to be processed. The function $Update\mathcal{G}Q$ (Algorithm S2) updates the graph $\mathcal{G}$ and the list Q based on the event numbers stored within $\mathcal{C}$. More precisely, $Update\mathcal{G}Q(\mathcal{G}, Q, (u, x, \mathbf{v}), m_1, m_2, e, \mathcal{C})$ searches all possibles $\mathbf{v}_1$ in $\mathcal{C}(m_1)$, and $\mathbf{v}_2$ in $\mathcal{C}(m_2)$ such that $\mathbf{v} = \mathbf{v}_1 \oplus \mathbf{v}_2 \oplus \mathbf{v}(e)$. For each pair of such vectors, a new event node of type e is added as a child of the node $(u, x, \mathbf{v})$ and this event node has $(m_1, \mathbf{v}_1)$ and $(m_2, \mathbf{v}_2)$ as children.

Algorithm S1 Constructing a minimum reconciliation graph from a event count vector matrix

1: **Input:** A dated subdivision species tree S', a gene tree G, a matrix of event count vectors $\mathcal{C}$.
2: **Output:** A reconciliation graph $\mathcal{G}$ that represents all reconciliations between G and S' associated to an event count vector contained in $\mathcal{C}$.
3: $Q \leftarrow$ an empty list of mapping nodes; *(the nodes to be explored)*;
4: $\mathcal{G} \leftarrow$ an empty reconciliation graph; *(the graph to populate)*;
5: **for** each node $x \in V(S')$ such that $\mathcal{C}(r(G), x) \neq \emptyset$ **do**
6:     **for** each $\mathbf{v} \in \mathcal{C}(r(G), x)$ **do**
7:         Add a node $(r(G), x, \mathbf{v})$ to $\mathcal{G}$ and Q; *(roots)*
8: **while** Q is not empty **do**
9:     Let $(u, x, \mathbf{v})$ be the head element of Q; Remove it from Q;
10:     **if** $u \in L(G), x \in L(S')$ and $s(u) = s(x)$ **then**
11:         **if** $(u, x, (0,0,0))$ is not in $\mathcal{G}$ **then** Add $(u, x, (0,0,0))$ to $\mathcal{G}$;
12:         Add to $\mathcal{G}$ an event node of type $\mathbb{C}$ having $(u, x, (0,0,0))$ as parent; *(leaves)*
13:     **else**
14:         **for** each e in $\{\mathbb{S}, \mathbb{T}, \mathbb{D}\}$ **do**
15:             **for** every $\{m_1, m_2\} \in postlist_e(u, x)$ **do**
16:                 $Update\mathcal{G}Q(\mathcal{G}, Q, (u, x, \mathbf{v}), m_1, m_2, e, \mathcal{C})$;
17:         **for** each e in $\{\varnothing, \mathbb{SL}, \mathbb{TL}\}$ **do**
18:             **for** every $m_1 \in postlist_e(u, x)$ **do**
19:                 $Update\mathcal{G}Q(\mathcal{G}, Q, (u, x, \mathbf{v}), m_1, \emptyset, e, \mathcal{C})$;
20: return $\mathcal{G}$;

Algorithm S2 $Update\mathcal{G}Q(\mathcal{G}, Q, (u, x, \mathbf{v}), m_1, m_2, e, \mathcal{C})$

1: **for** each $\mathbf{v}_1 = (d_1, t_1, l_1)$ in $\mathcal{C}(m_1)$ **do**
2:     $d_2 = d - d_1 - \mathbf{v}(e)_1$, $t_2 = t - t_1 - \mathbf{v}(e)_2$, $l_2 = l - l_1 - \mathbf{v}(e)_3$;
3:     $\mathbf{v}_2 := (d_2, t_2, l_2)$;
4:     **if** $\mathbf{v}_2 \in \mathcal{C}(m_2)$ or $\mathcal{C}(m_2) = \emptyset$ **then**
5:         **if** $(m_1, \mathbf{v}_1)$ is not in $\mathcal{G}$ **then** Add $(m_1, \mathbf{v}_1)$ to $\mathcal{G}$ and Q;
6:         Add an event node n_e of type e having $(u, x, \mathbf{v})$ as parent to $\mathcal{G}$;
7:         Add an edge from n_e to $(m_1, \mathbf{v}_1)$ to $\mathcal{G}$;
8:         **if** $\mathcal{C}(m_2) \neq \emptyset$ **then**
9:             **if** $(m_2, \mathbf{v}_2)$ is not in $\mathcal{G}$ **then** Add $(m_2, \mathbf{v}_2)$ to $\mathcal{G}$ and Q;
10:             Add an edge from n_e to $(m_2, \mathbf{v}_2)$ to $\mathcal{G}$;

Proof of Theorem 2

Correctness We first show that $\mathcal{G}$ is a reconciliation graph of G and S'. Property 1 in Definition 3 holds due to lines 5-7 of Algorithm S1. Now, let $m = (u, x, \mathbf{v})$ be a mapping node of $\mathcal{G}$. The first case of Property 2 holds due to line 7 of Algorithm 1 and lines $10 - 12$ of Algorithm S1. The second case of Property 2 holds because the function $Update\mathcal{G}Q$ used in Algorithm S1 never takes type $\mathbb{C}$ as an argument, thus we never create an event node of type $\mathbb{C}$ via this function. Moreover, the children set of m cannot be empty: in fact, m has been inserted in Q, and at least one event type is associated with m since we backtrack on the matrix $\mathcal{C}(u, x)$ computed by Algorithm 1. Now, let e be an event node of $\mathcal{G}$. Then e has always a unique parent due to the way $\mathcal{G}$ is updated in the function $Update\mathcal{G}Q$: we did not add any edge entering in an existent event node. If e is of type $\mathbb{C}$, then it is a leaf of $\mathcal{G}$ because we do not call the function $Update\mathcal{G}Q$ on its parent node. If e is not of type $\mathbb{C}$, then it must be a node created while calling the function $Update\mathcal{G}Q$ on its parent (mapping) node. From the calls to the function $Update\mathcal{G}Q$ at lines 16, 19, the remainder of Property 3 follows easily. Finally, it is easy to see that $\mathcal{G}$ is acyclic because, following Remark 1, possible cycles in $\mathcal{G}$ contain only $\mathbb{TL}$ events; but, again, a list of Pareto-optimal reconciliations cannot contain reconciliations with successive $\mathbb{TL}$ events. Therefore $\mathcal{G}$ is a reconciliation graph of G and S'.

Let T be a full subtree of $\mathcal{G}$. Since $\mathcal{G}$ is a reconciliation graph of G and S' as just shown, then Lemma 2 implies that T is a reconciliation tree of G and S', i.e. it depicts a reconciliation of G and S'. Moreover, the event count vector attributed to the root of T must be contained in one of $\mathcal{C}(r(G), \cdot)$, see lines $6-7$. Therefore T depicts a reconciliation of G and S' whose event count vector is contained in $\mathcal{C}(r(G), \cdot)$.

Let α be a reconciliation of G and S' such that $\mathbf{v}(\alpha)$ is contained in $\mathcal{C}(r(G), \cdot)$. Let T be the reconciliation tree that depicts α, we will prove that T is a full subtree of $\mathcal{G}$. We will prove that T is a subtree of $\mathcal{G}$ by recursion on the nodes of T via a pre-order traversal of T. By hypothesis $\mathbf{v}(\alpha)$ is contained in $\mathcal{C}(r(G), \cdot)$, hence the root of T has been added in to the graph $\mathcal{G}$ at lines 7-9. Let $m = (u, x, \mathbf{v})$ be a mapping node of T such that there exists the same mapping node in $\mathcal{G}$. Let e be the child of m in T. Suppose that e has two children $(u_1, y, \mathbf{v}_1)$ and $(u_2, z, \mathbf{v}_2)$. Then following Property 3 of Definition 3 $\{(u_1, y), (u_2, z)\} \in \mathit{postlist}_e(u, x)$ and $\mathbf{v} = \mathbf{v}(e) \oplus \mathbf{v}_1 \oplus \mathbf{v}_2$. Hence, the node e as well as the two mappings nodes $(u_1, y, \mathbf{v}_1)$, $(u_2, z, \mathbf{v}_2)$ and the associated edges must have been added to $\mathcal{G}$ by the function $\mathit{Update}\mathcal{G}Q$ at line 16. Similarly for the case that e has only one child. We continue this reasoning until the leaves of T, which must be also leaves of $\mathcal{G}$. In conclusion, T is a subtree of $\mathcal{G}$. Since T is a reconciliation tree of G and S', then T is a full subtree of $\mathcal{G}$.

The minimality of $\mathcal{G}$ can be justified using a similar reasoning as the one in Theorem 1 of [2].

Complexity The number of mapping nodes of the graph $\mathcal{G}$ returned by Algorithm S1 is $O(|V(S')| \times |V(G)|^3)$ because, for each $u \in V(G), x \in V(S')$ and $\mathbf{v} \in \mathcal{C}(u, x)$ there is at most one mapping node $(u, x, \mathbf{v})$, and $\mathcal{C}(u, x)$ has size $O(|V(G)|^2)$. Each mapping node $(u, x, \mathbf{v})$ can have $O(|V(G)|^2 \times |V(S)|)$ children of type $\mathbb{T}$, each one corresponding to a combination $\mathbf{v}_1, \mathbf{v}_2, z$ such that $\mathbf{v} = \mathbf{v}_1 \oplus +\mathbf{v}_2 \oplus (0, 1, 0)$, where $\mathbf{v}_1 \in \mathcal{C}(u_1, x)$, $\mathbf{v}_2 \in \mathcal{C}(u_2, z)$ and z is a node at time slice $\theta(x)$ different from x. The number of children of other event types are either $O(|V(S)|)$ (for $\mathbb{TL}$) or $O(1)$ (for all other types). Thus the number of nodes of $\mathcal{G}$ is $O(|V(G)|^5 \times |V(S)|^3)$ (note that $O(|V(S')|) = O(|V(S)|^2)$). It is also the size of the graph because there is no edge that links nodes of the same kinds.

For the running time, it is almost the same as Algorithm 3 in [2], except that: 1) the number of elements added in the list Q is $O(|V(G)|^3 \cdot |V(S')|)$ and not $O(|V(G)| \cdot |V(S')|)$; 2) the running time of the function $\mathit{Update}\mathcal{G}Q$ is $O(|V(G)|^2)$ and not constant as the function Update in [2]. Both functions use a hash function to check if a vector is in a given list or not. Hence, the complexity of Algorithm S1 is $O(|V(G)|^4)$ time the one of Algorithm 3 in [2], i.e. $O(|V(G)|^5 \cdot |V(S)|^3)$.

S1.4 Supplementary Experiments

S1.4.1 On the event supports computation by Xscape

Several options to compute event supports are proposed by Xscape. First, to generate the set of events associated to a region, option 'U' returns the list of all events that are present in *at least one* reconciliation of this region, while option 'I' returns only the events that are present in *all* reconciliations of this region. Second, the support of an event can be defined as the fraction of regions containing it or as the fraction of reconciliations containing it. However, the second case can only

be applied when using option 'I'. This is because *eventscape* only returns the total number of reconciliations for each region, and cannot – unlike our methods – return, for each event, the number of reconciliations containing it. Hence *eventscape* cannot make, among the events that are not present in all reconciliations – a distinction between the more frequent and the rare ones. Finally, if the region based support option is chosen, one can take into account the area of the region (the support is thus the fraction of event cost space containing the event). However, since in [1] the authors showed that using the area information can lead to biases, we did not test this support.

S1.4.2 **The accuracy on each event type**

We use the precision recall curves [3] to analyze the accuracy of each event type. Precision value is the fraction of recovered events that are true, and recall value is the fraction of true events that are recovered. A precision recall curve consists of points representing precision and recall values of a method. For example, a point $(0.1, 0.9)$ means that the method recovers 10% true events, and these true recovered events correspond to 90% of events estimated by this method. Hence, with the same recall value, the strategy giving a higher precision value is considered to be more accurate. Denote by $rD_s(t)$, $pD_s(t)$ respectively the recall and precision value of strategy s on duplication events after removing those that have supports smaller than t. We then have a precision and recall curve consisting of the points $(rD_s(t), pD_s(t))$ for $t = 0, \ldots, 100$ (Figures S1 $(a), (b)$. Similarly, we generate the precision and recall curves on transfer (Figures S1 $(c), (d)$) and loss events (Figures S1 $(e), (f)$) for each strategy. Note that increasing the threshold leads to a smaller set of estimated events which leads to a smaller recall value. In other word, the point at the right extremity of a curve corresponds to threshold 0, and the one at the left extremity corresponds to threshold 100.

In general, we see that, except for s6 and s7 using cost vector 1 on loss events, within a same strategy the smaller the recall is, the higher precision is. That shows the efficiency of these filtering strategies: increasing the threshold always leads to remove more false events than true events. However, their impacts, which are represented by the slopes of the curves, are not the same for each event type. Duplication curves have the highest slopes – and this means that filtering this type of event is the most efficient – followed by transfer curves and then loss curves.

Figures S1 $(a), (b)$ show that duplications are the most accurately estimated type of events for both cost vectors and for all of strategies. Both recall and precision values are very high for all strategies on this event type: about $60 - 80\%$ of true duplications were recovered, with a very high precision (about $80 - 100\%$).

Except strategies $6, 7$, loss events are the second accurately estimated events. Figures S1 $(c), (d)$ show that the recall values of strategies $1 - 5$ for losses are comparable to those of duplications (about 0.6-0.8), but with lower precision values. s6 and s7 work poorly in this case, and have "longer" curves than others, as we also observed in Figure 5. This result suggests that the poor performance of s6 and s7 is mainly due to the misplacement of loss events.

Figures S1 $(e), (f)$ show that transfer is the event type that is more difficult to recover in our experiments. The recall values are always smaller than 0.6 for all

methods, and with the same recall value, the corresponding precision value is much smaller than the one for other event type.

For completeness, we also give in Figure S2 the (FP, FN) curves of the 7 strategies on the two cost vectors. These curves are generated similarly as the curves in Figures 5 (c), (d) but for each event type: duplication, transfer, and loss. They have the same behavior as recall precision curves in figure S1, but not being normalized.

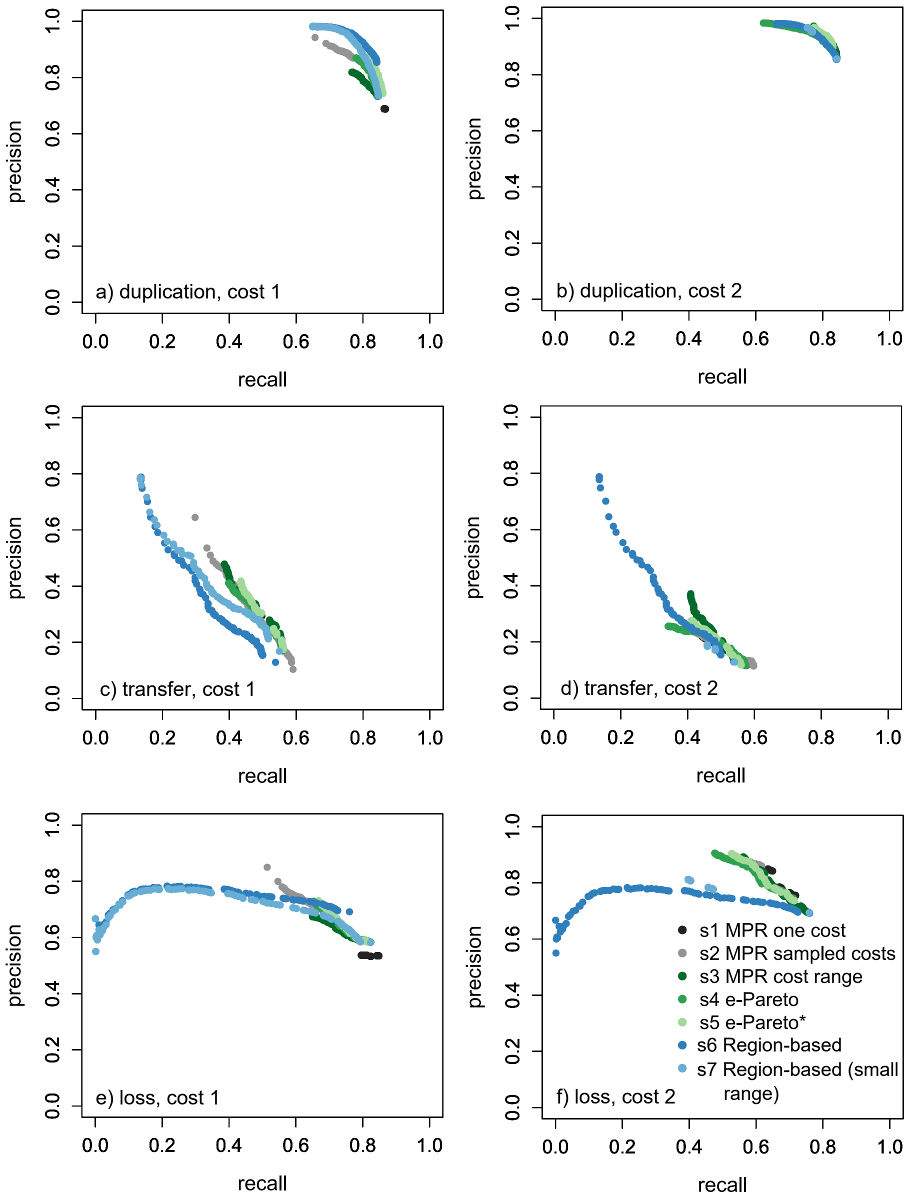

Figure S1 The precision and recall curves on each event type – duplication (a), (b), transfer (c), (d), and loss (e), (f) – for 7 strategies and two cost vectors described in the Experiments section. Each curve of strategy s for duplication events consists of the points $(rD_s(t), pD_s(t))$ where $rD_s(t)$ is the fraction of true duplications that are recovered by strategy s after removing those that have support smaller than t; and $pD_s(t)$ is the fraction of recovered duplications that are true by the same strategy. Transfer and loss curves are generated similarly.

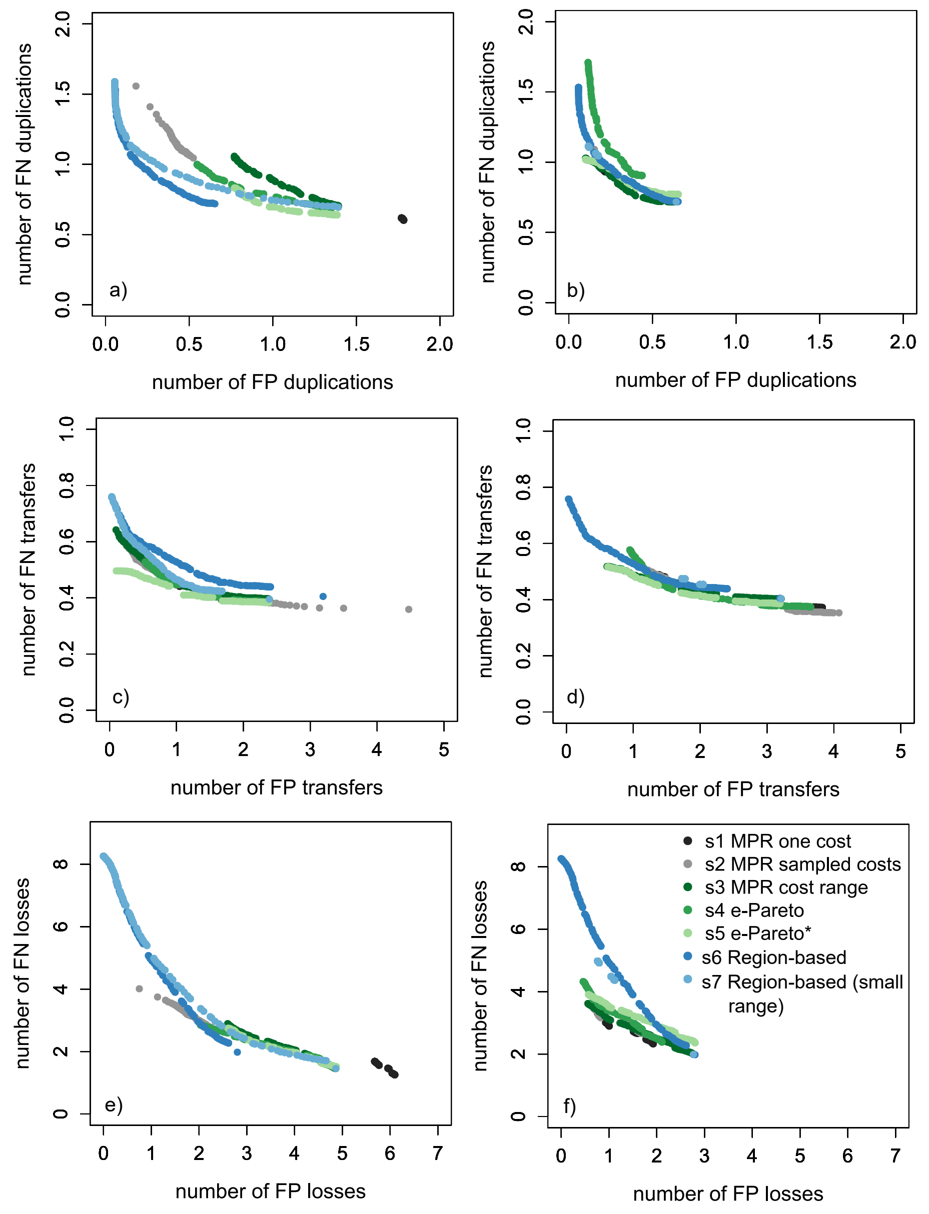

Figure S2 The (FP, FN) curves, which are generated as Figure 5 $(c), (d)$, but for each event type. Figures $(a), (c), (e)$ use cost vector 1, and Figures $(b), (d), (f)$ use cost vector 2.

Author details

References

1. Libeskind-Hadas, R., Wu, Y.-C., Bansal, M.S., Kellis, M.: Pareto-optimal phylogenetic tree reconciliation. Bioinformatics **30**(12), 87–95 (2014)
2. Scornavacca, C., Paprotny, W., Berry, V., Ranwez, V.: Representing a set of reconciliations in a compact way. J. Bioinformatics and Computational Biology **11**(2) (2013)
3. Davis, J., Goadrich, M.: The relationship between precision-recall and ROC curves. In: Proceedings of the 23rd International Conference on Machine Learning, pp. 233–240 (2006). ACM
